# Characterization of switchgrass (*Panicum virgatum* L.) PvKSL1 as a levopimaradiene/abietadiene‐type diterpene synthase

**DOI:** 10.1111/plb.13708

**Published:** 2024-08-20

**Authors:** G. Wyatt, P. Zerbe, K. Tiedge

**Affiliations:** ^1^ Department of Plant Biology University of California Davis USA; ^2^ Groningen Institute for Evolutionary Life Sciences, University of Groningen Groningen the Netherlands

**Keywords:** Bioenergy crops, diterpenoids, natural products, protein structure/function, specialized metabolism, terpene synthase

## Abstract

- The diverse class of plant diterpenoid metabolites serves important functions in mediating growth, chemical defence, and ecological adaptation. In major monocot crops, such as maize (*Zea mays*), rice (*Oryza sativa*), and barley (*Hordeum vulgare*), diterpenoids function as core components of biotic and abiotic stress resilience. Switchgrass (*Panicum virgatum*) is a perennial grass valued as a stress‐resilient biofuel model crop. Previously we identified an unusually large diterpene synthase family that produces both common and species‐specific diterpenoids, several of which accumulate in response to abiotic stress.
- Here, we report discovery and functional characterization of a previously unrecognized monofunctional class I diterpene synthase (PvKSL1) via *in vivo* co‐expression assays with different copalyl pyrophosphate (CPP) isomers, structural and mutagenesis studies, as well as genomic and transcriptomic analyses.
- In particular, PvKSL1 converts *ent*‐CPP into *ent*‐abietadiene, *ent*‐palustradiene, *ent*‐levopimaradiene, and *ent*‐neoabietadiene via a 13‐hydroxy‐8(14)‐*ent*‐abietene intermediate. Notably, although featuring a distinct *ent*‐stereochemistry, this product profile is near‐identical to bifunctional (+)‐levopimaradiene/abietadiene synthases occurring in conifer trees. PvKSL1 has three of four active site residues previously shown to control (+)‐levopimaradiene/abietadiene synthase catalytic specificity. However, mutagenesis studies suggest a distinct catalytic mechanism in PvKSL1. Genome localization of *PvKSL1* distant from other diterpene synthases, and its phylogenetic distinctiveness from known abietane‐forming diterpene synthases, support an independent evolution of PvKSL1 activity. Albeit at low levels, *PvKSL1* gene expression predominantly in roots suggests a role of diterpenoid formation in belowground tissue.
- Together, these findings expand the known chemical and functional space of diterpenoid metabolism in monocot crops.

## Graphical abstract

This study reports the discovery and functional characterization of PvKSL1, a previously unrecognized class I diterpene synthase from switchgrass.

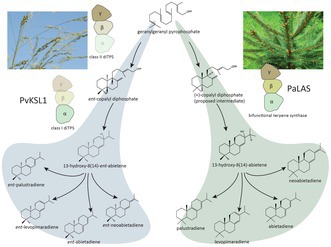

## INTRODUCTION

Diverse and dynamic networks of small‐molecule diterpenoid metabolites serve critical physiological functions in plant growth, defence, and environmental adaptation (Tholl 2015). Major monocot crops, such as maize (*Zea mays*), rice (*Oryza sativa*), wheat (*Triticum aestivum*), and barley (*Hordeum vulgare*), produce species‐specific blends of bioactive diterpenoids that have been demonstrated or predicted to function as core components of the chemical defence against pest and pathogen attack (Schmelz *et al*. 2014; Murphy & Zerbe 2020; Liu *et al*. 2021; Polturak *et al*. 2022). Beyond known functions in biotic stress responses, recent research supports a broader range of diterpenoid bioactivities, including roles in mediating abiotic stress responses and cooperative plant–microbe interactions (Vaughan *et al*. 2014, 2015; Christensen *et al*. 2018; Mafu *et al*. 2018; Murphy *et al*. 2021).

Known diterpenoids in monocot crops predominantly belong to the group of labdane‐type metabolites (Schmelz *et al*. 2014; Murphy & Zerbe 2020). Labdane chemical diversity is typically generated by the pairwise activity of class II and class I diTPSs, where class II diTPSs catalyse the transformation of the common 20‐carbon geranylgeranyl pyrophosphate (GGPP) precursor into bicyclic prenyl pyrophosphate compounds of distinct stereochemistry and oxygenation, and class I diTPSs facilitate the subsequent cleavage of the pyrophosphate group and various downstream cyclization and rearrangement reactions (Zi *et al*. 2014; Zerbe & Bohlmann 2015). Functional decorations of these diterpene scaffolds through the activity of cytochrome P450 monooxygenases, dehydrogenases, reductases, transferases, and other modifying enzyme classes define diterpenoid structural and functional diversity (Banerjee & Hamberger 2018; Bathe & Tissier 2019; Bryson *et al*. 2023). Beyond the functional variation within diTPS families, the ability of plants to combine catalytically distinct, monofunctional class II and class I diTPSs in the form of dynamic, modular metabolic networks further expands diterpenoid product diversity (Zi *et al*. 2014; Zerbe & Bohlmann 2015). These networks of monofunctional class II and class I diTPSs evolved through repeated events of gene duplication, domain loss, and neofunctionalization from archetypical bifunctional class II/I diTPSs that occur in select fungi, nonvascular plants and gymnosperm species (Peters *et al*. 2000; Schepmann *et al*. 2001; Martin *et al*. 2004; Hayashi *et al*. 2006; Ro & Bohlmann 2006; Cao *et al*. 2010; Mafu *et al*. 2011; Zerbe *et al*. 2012; Hall *et al*. 2013; Zi *et al*. 2014). In conifers, including species of fir (*Abies*), spruce (*Picea*), and pine (*Pinus*), these bifunctional enzymes form labdane‐type abietane and pimarane diterpenes that are further carboxylated to form diterpene resin acids as major components of chemical pest and pathogen defences (Keeling & Bohlmann 2006; Peters 2006; Geisler *et al*. 2016). As the key diTPSs in this defence mechanism, bifunctional levopimaradiene/abietadiene synthase (LAS) enzymes first convert GGPP into the bicyclic intermediate (+)‐copalyl diphosphate ((+)‐CPP), which is then transformed into abietadiene, levopimaradiene, palustradiene, and neoabietadiene via a hydroxylated 13‐hydroxy‐8(14)‐abietene intermediate (Peters *et al*. 2001; Keeling *et al*. 2008, 2011; Zerbe *et al*. 2012; Hall *et al*. 2013). While no bifunctional diTPSs occur in angiosperms, a few monofunctional class I diTPSs have been reported to convert different CPP stereoisomers into one or more LAS‐type abietane products, including, to current our knowledge, millet (*Setaria italica*) SiTPS8, wheat (*Triticum aestivum*) TaKSL2, marjoram (*Origanum majorana*) OmTPS5, and spurge (*Euphorbia peplus*) EpTPS8 and EpTPS23 (Zhou *et al*. 2012b; Johnson *et al*. 2019; Karunanithi *et al*. 2020). However, the downstream functional modifications and physiological functions of these metabolites remain largely unknown.

Switchgrass (*Panicum virgatum*; Poaceae) is a perennial C4 grass valued for its environmental resilience and agroeconomic importance as a lignocellulosic feedstock for biofuel production (Schmer *et al*. 2008). Prior studies in our group revealed a large diTPS gene family in the allotetraploid switchgrass (*P. virgatum* AP13) genome, which forms a complex biosynthetic network together with several characterized cytochrome P450 (CYP) enzymes, producing a diverse, species‐specific blend of labdane diterpenoids (Pelot *et al*. 2018; Tiedge *et al*. 2020). In addition to pimarane and manoyl oxide compounds also present in other monocot species, switchgrass produces unusual hydroxylated pimaranes and a group of furanoditerpenoids, termed panicoloids, that, to current knowledge, occur uniquely in switchgrass (Pelot *et al*. 2018; Muchlinski *et al*. 2021; Tiedge *et al*. 2022). Known panicoloids derive from *syn*‐CPP, 8,13‐CPP, and clerodienyl pyrophosphate (CLPP) through conversion by a group of P450s of the CYP71Z family (Muchlinski *et al*. 2021). Drought‐induced panicoloid accumulation in leaves and predominantly roots and demonstrated anti‐fungal properties *in vitro* suggest a role of panicoloids in switchgrass abiotic and biotic stress tolerance (Tiedge *et al*. 2022; Li *et al*. 2023).

Here, we report the discovery and biochemical characterization of *Pv*KSL1 as a multi‐substrate class I diTPS in switchgrass. *Pv*KSL1 shows promiscuity to different CPP isomers occurring in switchgrass and specifically catalyses the conversion of *ent*‐CPP into *ent*‐abietadiene, *ent*‐levopimaradiene, *ent*‐palustradiene, and *ent*‐neoabietadiene. Comparative mutational analysis of PvKSL1 suggests an independent evolution of the PvKSL1 function despite the conservation of key active site residues determining abietadiene, levopimaradiene, palustradiene, and neoabietadiene formation in bifunctional class II/I diTPS in several coniferous trees.

## METHODS

### Genomic and phylogenetic analyses

Genomic localization data for switchgrass *diTPS* genes were obtained from Phytozome (phytozome‐next.jgi.doe.gov). For phylogenetic analyses, peptide sequences were aligned via MUSCLE 3.8.425 (Edgar 2004) and a maximum likelihood phylogenetic tree was generated using the PhyML 3.3.20180621 plugin for Geneious Prime (Biomatters) with LG substitution model, four rate substitution categories, and 1000 bootstrap repetitions (Guindon *et al*. 2010). The phylogenetic comparison of PvKSL1 functional homologues is based on data and visualizations from Phytozome (phytozome‐next.jgi.doe.gov) and Archaepteryx.js (github.com/cmzmasek/archaeopteryx‐js), where the phylogenetic tree is inferred by FastTree2 based on approximate maximum likelihood (Price *et al*. 2010).

### Gene synthesis and site‐directed mutagenesis

An N‐terminally truncated (removal of 54 amino acids of a predicted plastidial transit peptide) and codon‐optimized *PvKSL1* gene (Table S2) was synthesized by TWIST Bioscience (USA) and cloned into the expression vector pETDuet‐1 (Novagen) for heterologous expression in *E. coli*. DNA synthesis and gene cloning were conducted in collaboration with the US Department of Energy Joint Genome Institute, a DOE Office of Science User Facility, under contract no. De‐AC02‐05CH11231. Single‐residue variants of PvKSL1 were generated using whole‐plasmid PCR amplification with site‐specific sense and anti‐sense oligonucleotides (Table S2) and Phusion HF Master Mix polymerase (New England Biolabs, USA). Restriction using *Dpn*1 was performed to remove parental plasmids before transformation into DH5α cells (Thermo Fisher, USA) for plasmid propagation. All protein variants were sequence‐verified before biochemical characterization.

### Enzyme functional analysis

For co‐expression functional analysis in *E. coli*, PvKSL1 was co‐transformed with a GGPP synthase from *Abies grandis* in a pACYCDuet vector and a pIRS plasmid carrying select upstream MEP pathway genes (Cyr *et al*. 2007; Morrone *et al*. 2010) into *E. coli* BL21DE3‐C43 cells (Lucigen, USA). Cultures were grown at 37 °C and 200 rpm in 50 ml Terrific Broth (TB) medium to OD_600_ 0.6 before cooling to 16 °C and induction of protein expression with 1 mm isopropyl‐β‐d‐1‐thiogalacto‐pyranoside (IPTG) (Genesee Scientific, USA) and the addition of 40 mm sodium pyruvate, and 1 mm MgCl_2_ (Murphy *et al*. 2019). After 72 h of incubation, metabolites were extracted with 50 ml hexane containing 0.35 ng·μl^−1^ 1‐Eicosene (Sigma‐Aldrich, USA) as internal standard. Extracts were air‐dried and then resuspended in 1 ml hexane for downstream analysis.

### GC–MS metabolite analysis

Enzyme products were analysed by GC‐MS on an Agilent 7890B GC device interfaced with a 5977 Extractor XL MS Detector at 70 eV electron ionization and 1.2 ml·min^−1^ He flow (Agilent, USA). Metabolite separation was performed using an Agilent DB‐5MS + DG column (30 m, 250 μm i.d., 0.25 μm film; Agilent) and the following GC parameters: 50 °C for 3 min, 15 °C·min^−1^ to 300 °C, hold for 4 min with pulsed splitless injection at 250 °C. MS data from 40 to 400 *m*/*z* were collected after a 13‐min solvent delay. Metabolites were either identified by comparison to authentic standards and reference mass spectra from Wiley NIST (Adams, 2007). In comparison to previous work by Keeling *et al*. (2011), who used cold on‐column injection and reduced oven temperatures to detect the thermally unstable alcohols with major ions of *m/z* 290 (parent ion), *m/z* 272 (loss of water [M‐18]), and *m/z* 247 (loss of an isopropyl group from the parent ion), the *m/z* 290 ion is still detectable at trace levels under our instrument configuration, possibly because of the high resolution of the GC‐MS instrument and increased abundance of the alcohol intermediates in the *E. coli* co‐expression system. Authentic standards of abietadiene, neoabietadiene, palustradiene, levopimaradiene, and 13‐hydroxy‐8(14)‐abietene were produced enzymatically using *Picea abies* levopimaradiene/abietadiene synthase (PaLAS; Martin *et al*. 2004). Metabolite quantification was based on three independent co‐expression experiments using 1‐Eicosene (Sigma‐Aldrich) as internal standard.

### Homology modelling and sequence analysis

A homology model of PvKSL1 was generated using the SWISS‐MODEL server (swissmodel.expasy.org) with the crystal structure of AgAS as template (PDB ID: 3S9V; Zhou *et al*. 2012a). The quality of the generated models was assessed based on Ramachandran plots with >95% (PvKSL1) and >97% (PaLAS) of residues in favoured regions, and QMEANDisCo quality scores of 0.7 (PvKSL1) and 0.89 (PaLAS). Protein sequence alignments were performed using the CLCBio Main Workbench (v.22.0; Qiagen, USA) with default parameters.

### RNA sequencing and gene expression analysis

Total RNA was extracted from 100 mg leaves or roots of both switchgrass ‘Alamo’ and ‘Cave‐in‐Rock’ varieties (*n* = 6) from control conditions and drought‐stressed conditions using a Monarch Total RNA Miniprep Kit (New England Biolabs, USA) and subsequently treated with DNase I for genomic DNA removal. Following assessment of RNA integrity and quantitation using the Bioanalyzer 2100 RNA Nano 6000 Assay Kit (Agilent Technologies), four of the six biological replicates with the highest RNA quality were selected for sequencing. Preparation of complementary DNA (cDNA) libraries and transcriptome sequencing was performed at Novogene (USA). In brief, following RNA integrity analysis and quantitation, cDNA libraries were generated using a NEBNext Ultra RNA Library Prep Kit (New England Biolabs) and sequenced on an Illumina Novaseq 6000 sequencing platform, generating 40–80 million 150 bp paired‐end reads per sample. Raw fastq file reads were filtered and trimmed using BBDuk, raw reads were evaluated for artefact sequence by kmer matching (kmer = 25), allowing one mismatch, and the detected artefact was trimmed from the 3′ end of the reads. RNA spike‐in reads, PhiX reads, and reads containing any N's were removed. Quality trimming was performed using the phred trimming method set at Q6. Finally, following trimming, reads below the length threshold were removed (minimum length 25 bases or 1/3 of the original read length; whichever is longer). Filtered, high‐quality reads were aligned to the reference genome (*P. virgatum* var. Alamo AP13 v.5.1) using HISAT2 (v2.2.0). For visualization, FPKM (Fragments Per Kilobase Million) values were log‐transformed and normalized. Before log transformation, a constant of 1 was added to the values to account for zeros in the data. Log‐transformed data was then standardized using z‐score normalization. Gene functional annotation was based on best matches to databases from Phytozome v13 (phytozome‐next.jgi.doe.gov), including *Arabidopsis*, rice, Gene Ontology (GO), and Panther, as well as in‐house protein databases of biochemically verified terpene metabolic enzymes (Pelot *et al*. 2018; Murphy & Zerbe 2020). Heatmaps were created using the pheatmap package in R (cran‐project.org, v.3.6.3). We utilized OpenAI's GPT‐4 model for guidance in data processing and visualization.

## RESULTS

### Genomic and phylogenetic analyses

**Fig. 1:**
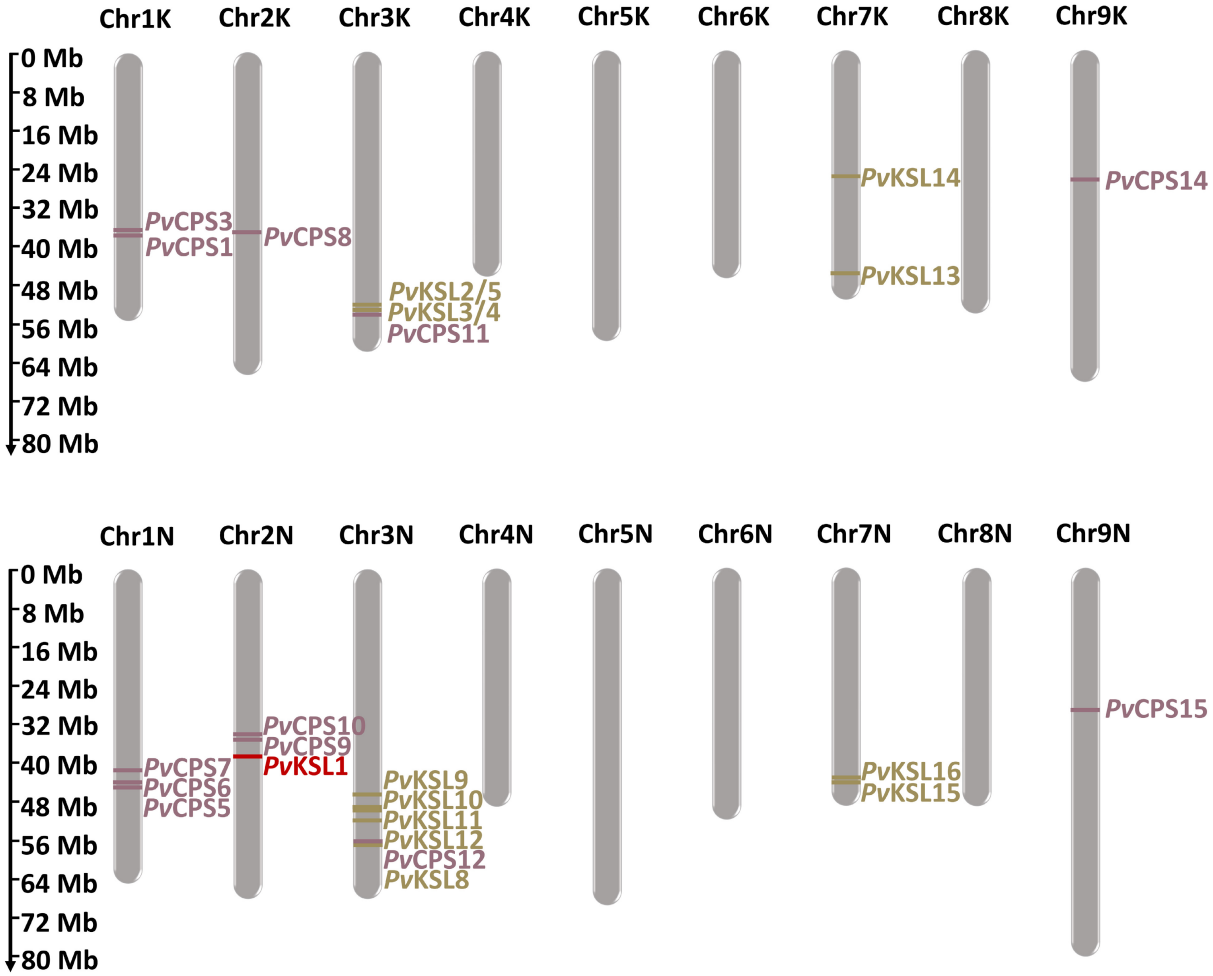
Chromosome localization of characterized diterpene synthases (diTPS) across the K (top) and N (bottom) switchgrass subgenomes. Class II diTPSs that catalyse the protonation‐initiated cyclization of geranylgeranyl pyrophosphate (GGPP) are labelled as copalyl diphosphate synthases (CPS) in purple; class I diTPSs that catalyse the ionization‐initiated cyclization and rearrangement reactions are labelled as kaurene synthase‐like (KSL) in brown; PvKSL1 is labelled in red; chromosome ideogram built with MG2C (Chao *et al*. 2021).

The gene *Pv*KSL1 was identified by mining the allotetraploid *P. virgatum* (v5.1; Lovell *et al*. 2021) using an established gene discovery pipeline (Zerbe *et al*. 2013; Tiedge *et al*. 2020). Sequence analysis showed the DDxxD divalent metal‐binding motif in the ɑ‐domain and absence of a DxDD motif in the N‐terminal active site in the γβ‐domain to classify *Pv*KSL1 as a three‐domain class I diTPS. *PvKSL1* is located on chromosome 2 of the N subgenome, with approximately 4 Mb distance from two class II diTPSs, *Pv*CPS9 and *PvCPS10*, that have been characterized and predicted to function as *syn*‐CPP synthases, respectively (Fig. 1).

**Fig. 2:**
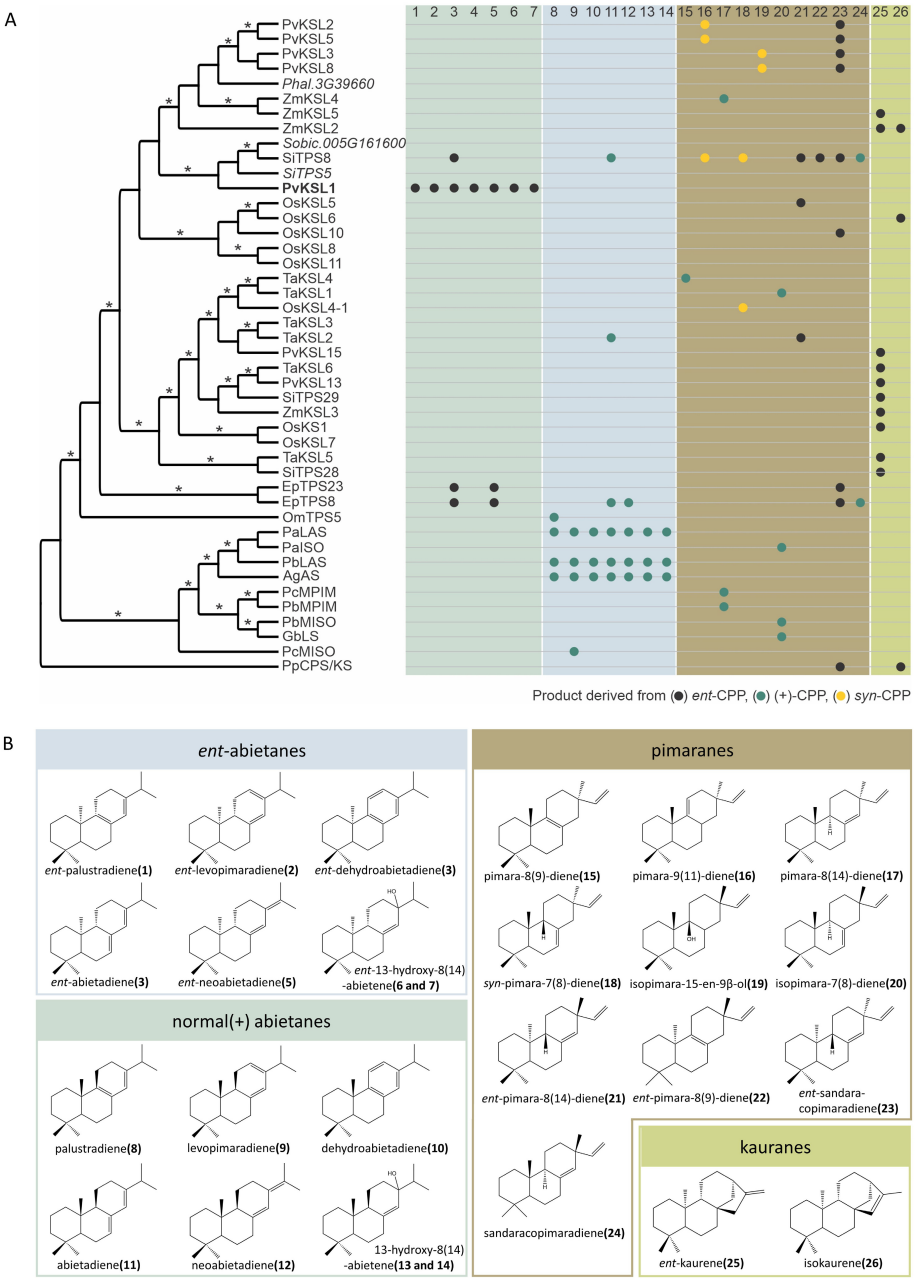
Distribution and diversity of abietane‐ and pimarane‐biosynthetic diterpene synthases. (A) Maximum‐likelihood phylogenetic tree of known class I diTPS. Pv = *Panicum virgatum*; Phal = *Panicum hallii*; Zm = *Zea mays*; Sobic = *Sorghum bicolor*; Si = *Setaria italica*; Os = *Oryza sativa*; Ta = *Triticum aestivum*; Ep = *Euphorbia peplus*; Om = *Origanum majorana*; Pa = *Picea abies*; Pb = *Picea banksiana*; Ag = *Abies grandis*; Pc = *Pinus contorta*; Gb = *Ginkgo biloba*; Pp = *Physcomitrella patens*. GenBank Accession numbers are listed in Table S1; bootstrap values of ≥85% (1000 repetitions) are shown. (B) Product structures of enzymes included in the phylogenetic tree.

Phylogenetic analysis of PvKSL1 and known diTPSs showed a broad distribution of abietane‐forming enzymes across different monocot and dicot species, in addition to the branch of bifunctional class II/I diTPSs in gymnosperms (Fig. 2A). PvKSL1 is placed within a separate branch comprised of two uncharacterized diTPSs from *S. italica* and *Sorghum bicolor*, as well as *S. italica* SiTPS8 that has high substrate promiscuity converting (+)‐CPP, *ent*‐CPP, or *syn*‐CPP into several diterpenoid scaffolds including (+)‐ and *ent*‐abietadiene (Karunanithi *et al*. 2020). Other characterized *ent*‐abietane‐forming diTPS, EpTPS8 and EpTPS23 from *Euphorbia peplus* (Andersen‐Ranberg *et al*. 2016) are phylogenetically distant from PvKSL1 (Fig. 2A, B). Additionally, the phylogenetically more distant wheat (*Triticum aestivum*) diTPS TaKSL2 produces (+)‐abietadiene (**11**) from (+)‐CPP and *ent*‐pimara‐8(14),15‐diene (**21**) from *ent*‐CPP (Zhou *et al*. 2012b). Within the Lamiaceae, marjoram (*Origanum majorana*) OmTPS5 can form (+)‐palustradiene (Johnson *et al*. 2019).

**Fig. 3:**
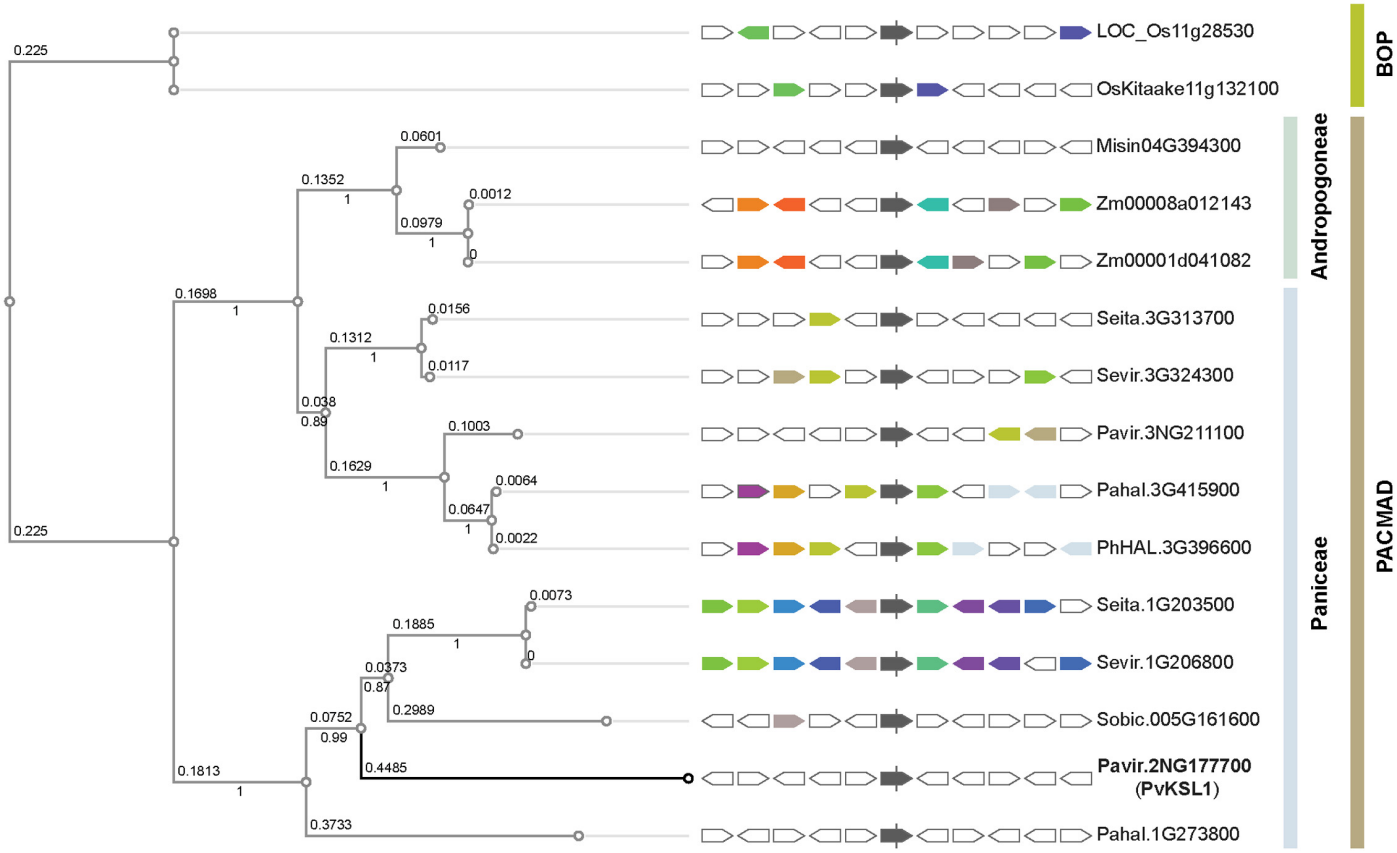
Phylogenetic tree of PvKSL1 and functionally related genes with upstream and downstream systemic neighbours. Included homologous enzymes were identified via hmmersearch (HMMER, EMBL‐EBI) based on functional annotations/protein domains. *PvKSL1* and homologues are centrally displayed in dark grey, with the five closest up‐ and downstream genes sharing colours based on homologies. Gene IDs, data, and visualization partially derived with permission from Phytozome v13. PACMAD = clade including the Panicoideae, Arundinoideae, Chloridoideae, Micrairoideae, Aristidoideae, and Danthonioideae subfamilies; BOP = clade including the Bambusoideae, Oryzoideae, and Pooideae subfamilies; Os = *Oryza sativa*; Misin = *Miscanthus sinensis*; Zm = *Zea mays*; Seita = *Setaria italica*; Sevir = *Setaria viridis*; Pavir = *Panicum virgatum*; Pahal and PhHAL = *Panicum hallii*; Sobic = *Sorghum bicolor*.

To investigate the evolution of PvKSL1, we next compared its protein sequence to homologues from other Poaceae. The subfamilies Panicoideae, Arundinoideae, Chloridoideae, Micrairoideae, Aristidoideae, and Danthonioideae, referred to as the PACMAD clade, represent the lineages of Poaceae in which C4 photosynthesis has evolved in grasses. We selected agriculturally relevant representatives of the PACMAD clade, including *Panicum hallii*, *S. bicolor*, *Z. mays*, *S. italica*, *S. viridis*, and *Miscanthus sinensis* for sequence comparison. The sister group, termed BOP clade, includes the subfamilies Bambusoideae, Oryzoideae, and Pooideae, which contain grasses with C3 photosynthesis. From this clade, we selected *O. sativa* to be included in the sequence analysis (Fig. 3). As expected, the phylogenetically more distant rice homologues branch furthest away, while within the PACMAD clade two major branches form. One branch contains the more conserved group of homologues with slower mutational rates (indicated by shorter branch length). The other branch contains PvKSL1 with the highest number of mutations as well as other homologues that experienced a higher number of evolutionary changes.

**Fig. 4:**
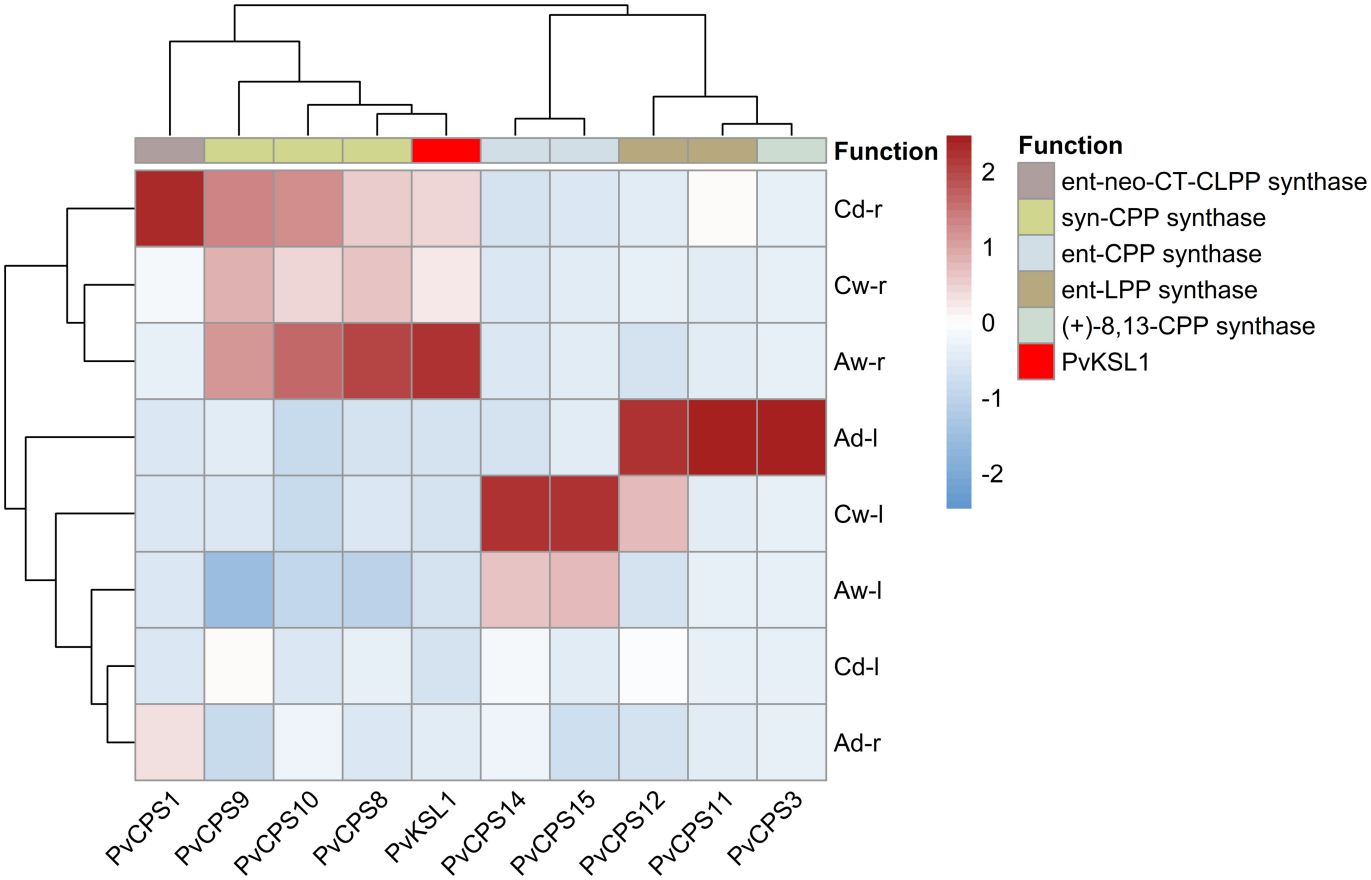
Relative expression of *PvKSL1* with characterized switchgrass class II diterpene synthases via hierarchical clustering of z‐scored and log‐transformed RNA‐seq FPKM values. C = ‘Cave‐in‐Rock’; A = ‘Alamo’; d = drought‐stressed; w = well‐watered; r = roots; l = leaves.

Publicly available RNAseq data (Phytozome GeneAtlas v2) showed expression of *PvKSL1* primarily in switchgrass roots (Figure S1). In addition to the publicly available data, we exposed two switchgrass varieties from different ecotypes, the lowland ecotype ‘Alamo’ and the upland ecotype ‘Cave‐in‐Rock’, to either terminal drought or well‐watered conditions and extracted RNA from both leaf and root tissue for Illumina RNA sequencing. Analysis of the transcriptome data supported root‐specific expression of *PvKSL1* in ‘Cave‐in‐Rock’ under well‐watered and drought‐stressed conditions, and especially in ‘Alamo’ control plants (Fig. 4). Furthermore, hierarchical co‐expression analysis of *PvKSL1* with other characterized switchgrass diTPSs shows significant co‐expression of *PvKSL1* with the *syn*‐CPP synthases *PvCPS8*, *PvCPS9*, and *PvCPS10*.

### Functional characterization

**Fig. 5:**
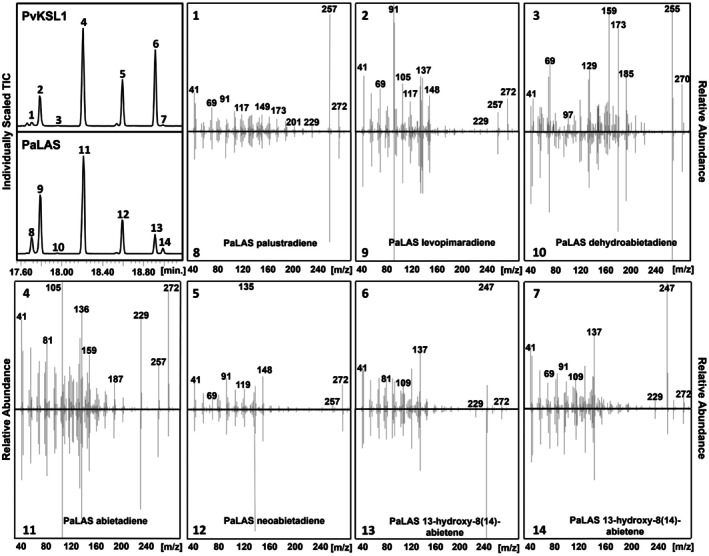
PvKSL1 product profile in comparison to PaLAS. GC–MS chromatogram and corresponding mass spectra of the extracted PvKSL1 products compared to the PaLAS‐derived abietanes: *ent*‐palustradiene (1); *ent*‐levopimaradiene (2); *ent*‐dehydroabietadiene (3); *ent*‐abietadiene (4); *ent*‐neoabietadiene (5); *ent*‐13‐hydroxy‐8[14]‐abietane isomers (6 + 7); (+)‐palustradiene (8); (+)‐levopimaradiene (9); (+)‐dehydroabietadiene (10); (+)‐abietadiene (11); (+)‐neoabietadiene (12); (+)‐13‐hydroxy‐8[14]‐abietane isomers (13 and 14).

To assess the biochemical function of *Pv*KSL1, we performed *in vivo* co‐expression studies using an *E. coli* platform engineered for diterpenoid production (Cyr *et al*. 2007; Morrone *et al*. 2010). PvKSL1 was individually paired with all characterized switchgrass class II diTPS: *ent*‐CPP synthase, *ent*‐LPP synthase, *syn*‐CPP synthase, *ent‐neo*‐CT‐CLPP synthase, and 8‐13‐CPP synthase (Pelot *et al*. 2018). GC–MS analysis of the extracted enzyme products determined that *Pv*KSL1 forms products with *ent*‐CPP, *syn*‐CPP, and *ent*‐LPP, but not *ent‐neo*‐CT‐CLPP or 8,13‐CPP (Fig. 5, Figures S2–S5). PvKSL1 produced eight pimarane‐type compounds (a‐h) from *syn*‐CPP, although in lower amounts compared to the products derived from *ent*‐CPP (Figures S2 and S7). Based on mass spectral library best matches, we tentatively identified these compounds as double‐bond isomers of pimaradiene. PvKSL1 also produced abietane‐type compounds (i) and (j) from *ent*‐LPP, but only in trace amounts (Figure S3). Low abundance of products (a‐j) prevented further structural identification via NMR analysis. PvKSL1‐catalysed conversion of *ent*‐CPP resulted in four major products identified as *ent*‐palustradiene (**1**), *ent*‐levopimaradiene (**2**), *ent*‐abietadiene (**4**), and *ent*‐neoabietadiene (**5**), based on comparison to the product profile of *Picea abies* levopimaradiene/abietadiene synthase (PaLAS; Fig. 5). Note that the PaLAS products are derived from (+)‐CPP but both enantiomers co‐elute under the chosen GC‐MS conditions. In addition, both isomers of *ent*‐13‐hydroxy‐8(14)‐abietene (**6** and **7**) were identified which had previously been shown to function as direct precursors to the PaLAS diterpene olefin products (Keeling *et al*. 2011).

To further investigate the substrate promiscuity of PvKSL1, we tested the activity of *Pv*KSL1 with (+)‐CPP as a substrate that, to current knowledge, is not produced in switchgrass. *E. coli* co‐expression with the maize (+)‐CPP synthase ZmCPS3 (Murphy *et al*. 2018) did not yield the (+)‐stereoisomers of levopimaradiene, palustradiene, abietadiene, and neoabietadiene, but trace amounts of diterpene olefins that could not be definitely identified (Figure S6).

### Structure–function analysis of PvKSL1

**Fig. 6:**
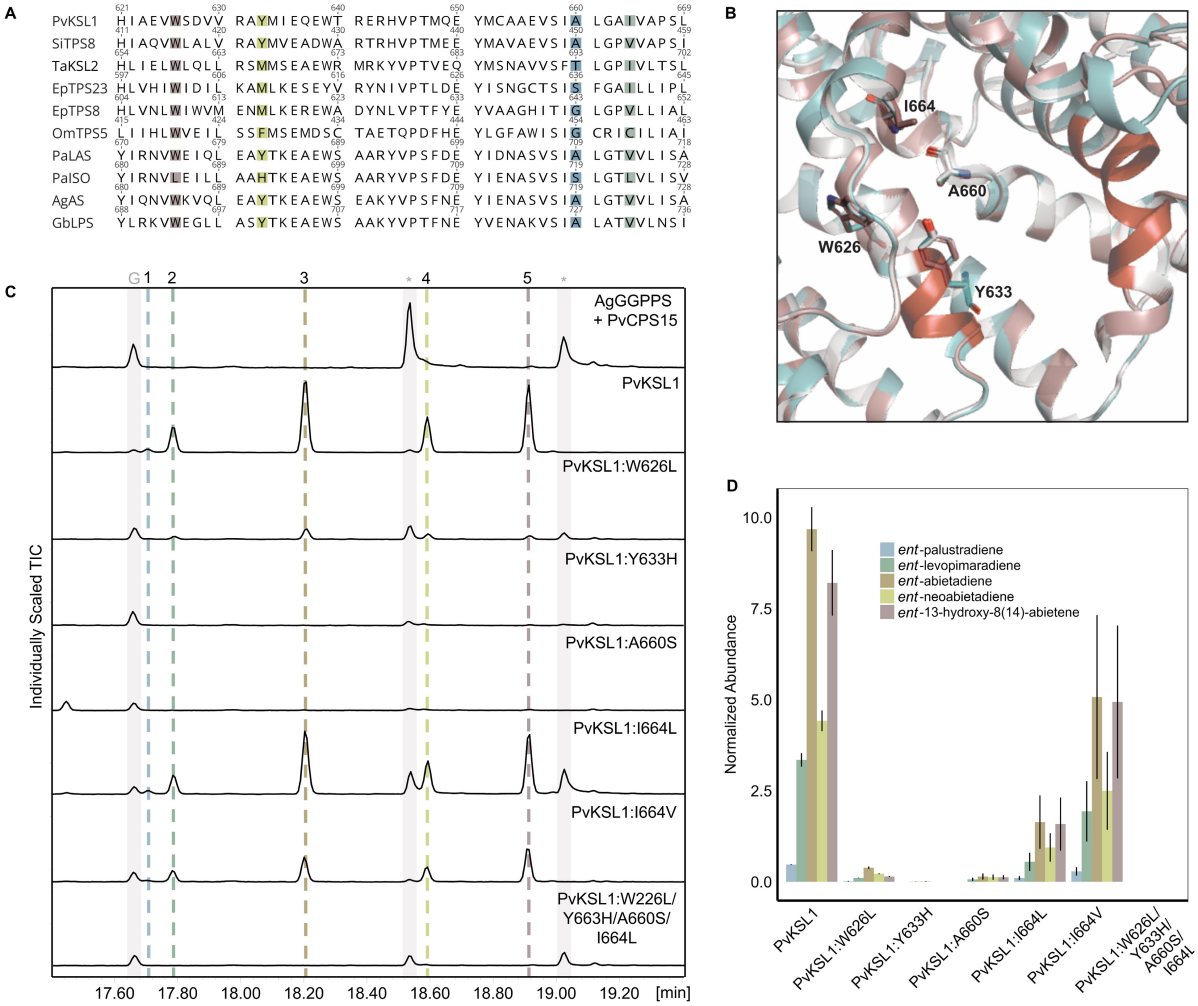
Structure–function analysis of PvKSL1. (A) Amino acid alignment of class I active site residues of PvKSL1 as compared to diterpene synthases previously shown to produce variations of abietadiene, levopimaradiene, palustradiene, and/or neoabietadiene. Asterisks indicate residues targeted for mutagenesis in this study. Pv = *Panicum virgatum*; Ta = *Triticum aestivum*; Si = *Setaria italica*; Ep = *Euphorbia peplus*; Om = *Origanum majorana*; Pa = *Picea abies*; Ag = *Abies grandis*; Gb = *Ginkgo biloba*; TPS = terpene synthase; LAS = levopimaradiene/abietadiene synthase; ISO = isopimaradiene synthase. (B) Superposition of homology models of PvKSL1 (blue) and *Picea abies* LAS (PaLAS, brown) as based on the crystal structure of *Abies grandis* abietadiene synthase (AgAS, Protein Data Bank code: 3S9V). Residues targeted for site‐directed mutagenesis in this study are depicted as stick models. The active site DDxxD and NSE/DTE motifs are shown in red. (C) GC‐MS chromatogram of PvKSL1 wild‐type and protein variants. The grey bars highlight geranylgeranyl pyrophosphate (GGPP; RT 17.66 min) and *ent*‐CPP (PvCPS15; RT 18.52 min). (D) Normalized relative abundance of PvKSL1 products for wild‐type and protein variants.

Having identified PvKSL1 as a diTPS producing a product profile that is, albeit with distinct *ent*‐stereochemistry, highly similar to that of conifer LAS‐type bifunctional diTPSs, we next performed comparative sequence and structural analyses to investigate the underlying structure–activity relationships. Previous research demonstrated four active site positions that control product specificity in PaLAS (W679L, Y686, A713, V717) and the closely related *P. abies* isopimaradiene synthase PaISO (L687, H694, S721, L725) (Keeling *et al*. 2008). Sequence comparison of PvKSL1 with PaLAS, PaISO and other known class I and class II/I diTPS that form abietadiene, palustradiene, sandaracopimaradiene or neoabietadiene showed varying degrees of conservation at these four active site positions. Specifically, despite only 27% protein sequence identity of the α‐domain containing the class I active site in PvKSL1 and PaLAS, the PvKSL1 active site features conserved W626, Y633, and A660 residues at the respective positions and differs from PaLAS only in the fourth residue containing an I664 instead of a V residue (Fig. 6A). Homology modelling of PvKSL1 based on the crystal structure of the *Abies grandis* abietadiene synthase (Zhou *et al*. 2012a) confirmed that PvKSL1 adopts the common βγα‐domain fold typical for class I diTPS, featuring the conserved DDxxD and NSE/DTE substrate‐binding motifs in the C‐terminal active site. The positions of the W626, Y633, A660, and I664 active site residues showed no significant difference to the PaLAS active site contour (Fig. 6B).

To assess the catalytic relevance we used site‐directed mutagenesis to generate PvKSL1 variants that match the PaLAS catalytic residues (PvKSL1:I664V) as well as the PaISO active site, namely PvKSL1:W626L, PvKSL1:Y664H, PvKSL1:A660S, PvKSL1:I664L, and PvKSL1:W626L/Y633H/A660S/I664L (Fig. 6A). *E. coli* co‐expression assays of the tested PvKSL1 variants, as compared to wild‐type PvKSL1, showed overall reduced catalytic activity with variants PvKSL1:Y664H, PvKSL1:A660S, and the quadruple variant PvKSL1:W626L/Y633H/A660S/I664L showing a near‐complete loss of function (Fig. 6C, D). No change in PvKSL1 product specificity, as previously observed in variants of PaLAS (Keeling *et al*. 2008) was identified for any of the single or quadrupole variants.

## DISCUSSION

**Fig. 7:**
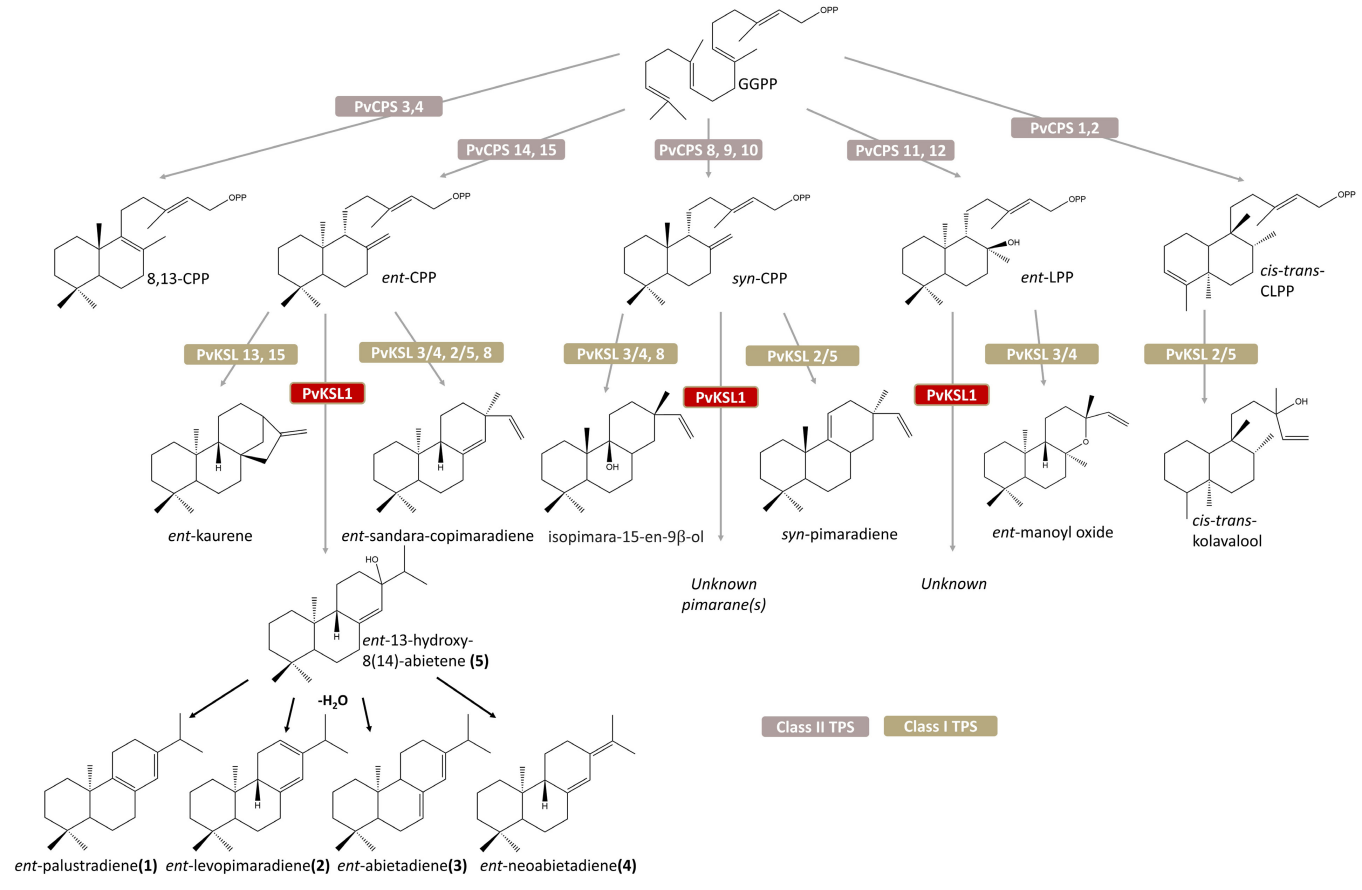
Known diterpenoid biosynthetic pathways in switchgrass. The central diterpenoid precursor, geranylgeranyl pyrophosphate (GGPP) is cyclized by class II diterpene synthases (diTPSs) to form prenyl pyrophosphate intermediates of distinct stereochemistry and oxygenation. Class I diTPSs, which are often promiscuous, convert the prenyl pyrophosphate intermediates through cleavage of the pyrophosphate group and downstream rearrangements into a range of diterpene scaffolds. These scaffolds can then be further functionally modified by cytochrome P450 monooxygenases (P450s) to form various diterpenoids in both general (gibberellin phytohormone) and specialized diterpenoid metabolism.

The early evolutionary divergence of the plant diTPS family and the ability of these enzymes to function in modular biosynthetic networks have given rise to a vast chemical space of bioactive diterpenoids (Zi *et al*. 2014; Zerbe & Bohlmann 2015; Wang *et al*. 2023). Given the physiological importance of diterpenoids as phytohormones and specialized metabolites that serve as core components of plant chemical defence mechanisms and other environmental interactions (Gershenzon & Dudareva 2007; Schmelz *et al*. 2014; Tholl 2015), investigating the diversity and biosynthesis of diterpenoids might potentially contribute to developing new strategies for crop optimization through precision breeding and crop engineering (Schmelz *et al*. 2014; Jez *et al*. 2016; Murphy & Zerbe 2020). The discovery and functional characterization of PvKSL1 as a multi‐functional class I diTPS, which can act in a pairwise reaction with different switchgrass class II diTPSs, producing *ent*‐CPP, *syn*‐CPP, and *ent*‐LPP, to form a range of pimarane and abietane diterpenoids, adds a previously unrecognized enzyme to the large and diverse diterpenoid metabolic network in switchgrass (Fig. 7).

Notably, no *PvKSL1* homologue was identified in the K subgenome, suggesting that a possible gene loss in the K subgenome or a gain‐of‐function event after the genome duplication resulting in the allotetraploid *P. virgatum* genome gave rise to *PvKSL1* on the N subgenome. Co‐localization of *PvKSL1* with the characterized *syn*‐CPP synthases *PvCPS9* and *PvCPS10* (Pelot *et al*. 2019) (Fig. 1), as well as patterns of co‐expression with *PvCPS9*, *PvCPS10*, and the functionally characterized *syn*‐CPP synthase *PvCPS8* (Pelot *et al*. 2019) (Fig. 4), suggests a role for PvKSL1 in the formation of *syn*‐CPP‐derived diterpenoids. Biosynthesis of *syn*‐pimarane diterpenoids by co‐expressing PvCPS8 and PvKSL1 supports such a function. However, pairwise activity of PvCPS8 and PvKSL1 resulted in lower product amounts as compared to the product abundance observed when combining PvKSL1 with the *ent*‐CPP synthase PvCPS15 (Figure S7). Therefore, it appears plausible that PvKSL1 catalyses the committed reaction to different pathway branches, possibly depending on condition‐specific co‐expression and activity with different class II diTPSs. Furthermore, the enzyme assays were performed in a heterologous system and therefore the catalytic activity might differ from the endogenous capacity in switchgrass.

### PvKSL1 has potentially developed via convergent evolution

Prior to this study, knowledge of monocot diTPSs forming abietane diterpenoids included SiTPS8 from *Setaria italica* (Karunanithi *et al*. 2020) and TaKSL2 from *Triticum aestivum* (Zhou *et al*. 2012b), which convert (+)‐CPP into sandaracopimaradiene and abietadiene, respectively. Similar to PvKSL1, SiTPS8 and TaKSL2 also show substrate promiscuity, converting *ent*‐CPP and *syn*‐CPP into different pimarane and abietane scaffolds (Zhou *et al*. 2012b; Karunanithi *et al*. 2020; Polturak *et al*. 2022). The fast mutation rate of PvKSL1 in comparison to other enzymes related at a functional domain level (Fig. 3) supports the possibility that the specialized functionality of PvKSL1 to catalyse abietane biosynthesis has independently arisen via convergent evolution. While most TPSs are considered to have evolved divergently (Jia *et al*. 2022), individual appearances of convergent evolution have been observed in other plants (Trapp & Croteau 2001; Aubourg *et al*. 2002; Sharkey *et al*. 2005). The functional plasticity and genetic multiplicity observed in PaLAS, PvKSL1, and other TPSs support evolution through convergent metabolic evolution. Additionally, in the case of switchgrass, the possibility of independent evolution aligns with the emergence of other abiotic and biotic stress tolerance traits that have independently evolved in C4 grasses (Pardo & VanBuren 2021).

### Active site residues are not responsible for product specificity but for catalytic activity

Previous research on gymnosperm class II/I diTPSs identified four active site residues that largely control product specificity in the biosynthesis of palustradiene, levopimaradiene, abietadiene, and neoabietadiene (Peters & Croteau 2002; Wilderman & Peters 2007; Keeling *et al*. 2008). Conservation of three of these residues in PvKSL1 suggested a possibly similar mechanism. However, mutation analysis of PvKSL1 demonstrated that all residues are important for catalytic activity, yet they do not impact product specificity, suggesting that other active site residues impart this function. The PvKSL1‐catalysed conversion of (+)‐CPP not into palustradiene, levopimaradiene, abietadiene, or neoabietadiene but into other so far unidentified labdane diterpenoids also supports a different enzyme mechanism.

### Root‐specific expression suggests a role in belowground interactions

Root‐specific expression of *PvKSL1* in both public and in‐house transcriptome data is aligned with the biosynthesis and accumulation of pimarane‐type and panicoloid root diterpenoids previously identified in switchgrass (Muchlinski *et al*. 2021; Tiedge *et al*. 2022), and may suggest roles in belowground plant–environment interactions, as shown for root diterpenoids in rice (Kato‐Noguchi & Peters 2013; Schmelz *et al*. 2014), maize (Schmelz *et al*. 2011, 2014; Vaughan *et al*. 2014; Mafu *et al*. 2018; Ding *et al*. 2019), and barley (Liu *et al*. 2021). However, unlike panicoloid diterpenoids that accumulate in root tissue in response to drought stress (Tiedge *et al*. 2022), *PvKSL1* did not show drought‐inducible gene expression, and no PvKSL1 products were detected in switchgrass tissues. Considering that the vast majority of plant diTPS products are further functionally modified, it is plausible that the PvKSL1 products are rapidly metabolized and, thus, not detectable *in planta*. Future pathway and metabolite discovery efforts will be required to gain a deeper understanding of the dynamic regulation and function of PvKSL1‐derived pathway branches and their products in switchgrass stress responses.

## AUTHOR CONTRIBUTIONS

GW and KT carried out the genomic, transcriptomic and metabolomic analyses as well as the functional enzyme characterization. GW performed the mutagenesis experiments. PZ and KT contributed to the design and implementation of the study and analysis of the results. All authors wrote and approved the final version of the manuscript.

## FUNDING INFORMATION

Financial support for this work was provided by an NSF PGR TrTECH award (2312181) to PZ, a German Research Foundation (DFG) Research Fellowship (TI 1075/1‐1) to KT, an NSF Graduate Research Fellowship (2036201) to GW, and the DOE Joint Genome Institute (JGI) DNA Synthesis Science Program (grant #2568) to PZ. The gene synthesis work conducted by the U.S. Department of Energy Joint Genome Institute (JGI), a DOE Office of Science User Facility, is supported by the Office of Science of the U.S. Department of Energy under Contract No. DE‐AC02‐05CH11231.

## Supporting information


**Figure S1.** Hierarchical clustering of *Pv*KSL1 with other characterized diTPS using publicly available transcriptome data; all metadata can be found on GeneAtlas (phytozome‐next.jgi.doe.gov/geneatlas).
**Figure S2.** GC‐MS chromatogram and mass spectra of product profiles for switchgrass *syn*‐CPP synthase (PvCPS8) alone and in combination with PvKSL1, resulting in unknown products (a–h).
**Figure S3.** GC‐MS chromatogram and mass spectra of product profiles for switchgrass *ent*‐LPP synthase (PvCPS11) alone and in combination with PvKSL1, resulting in unknown products (i) and (j).
**Figure S4.** GC‐MS chromatogram of product profiles for switchgrass *ent‐neo‐CT‐CLPP* synthase alone and in combination with PvKSL1; no new products resulting.
**Figure S5.** GC‐MS chromatogram of product profiles for switchgrass 8,13‐CPP synthase alone and in combination with PvKSL1; no new products resulting.
**Figure S6.** GC‐MS chromatogram and mass spectra of product profiles for *Zea mays* (+)‐CPP synthase (*Zm*CPS3) alone and in combination with *Pv*KSL1, resulting in unknown products (k), (l), (m), and (n).
**Figure S7.** GC‐MS chromatogram of product profiles for PvKSL1 with *syn*‐CPP synthase (PvCPS8) (black) and *ent*‐CPP synthase (PvCPS15) (red) showing relative abundance.
**Table S1.** GenBank accession numbers for Fig. 2.
**Table S2.** Nucleotide information of gene sequences used in this study.

## Data Availability

The RNA sequencing data were submitted to the Sequence Read Archive (SRA; ncbi.nlm.nih.gov/sra), accession no. PRJNA1038876 – PRJNA1038863.
