# Supplementary material for: Characterization of switchgrass (Panicum virgatum L.) PvKSL1 as a levopimaradiene/abietadiene‐type diterpene synthase
Source: Plant Biol (Stuttg). 2024 Aug 20;27(5):698–709. doi: 10.1111/plb.13708 (PMC12255283; doi:10.1111/plb.13708)
Supplement: Supplementary file 1 — Figure S1. Hierarchical clustering of PvKSL1 with other characterized diTPS using publicly available transcriptome data; all metadata can be found on GeneAtlas (phytozome‐next.jgi.doe.gov/geneatlas). Figure S2. GC‐MS chromatogram and mass spectra of product profiles for switchgrass syn‐CPP synthase (PvCPS8) alone and in combination with PvKSL1, resulting in unknown products (a–h). Figure S3. GC‐MS chromatogram and mass spectra of product profiles for switchgrass ent‐LPP synthase (PvCPS11) alone and in combination with PvKSL1, resulting in unknown products (i) and (j). Figure S4. GC‐MS chromatogram of product profiles for switchgrass ent‐neo‐CT‐CLPP synthase alone and in combination with PvKSL1; no new products resulting. Figure S5. GC‐MS chromatogram of product profiles for switchgrass 8,13‐CPP synthase alone and in combination with PvKSL1; no new products resulting. Figure S6. GC‐MS chromatogram and mass spectra of product profiles for Zea mays (+)‐CPP synthase (ZmCPS3) alone and in combination with PvKSL1, resulting in unknown products (k), (l), (m), and (n). Figure S7. GC‐MS chromatogram of product profiles for PvKSL1 with syn‐CPP synthase (PvCPS8) (black) and ent‐CPP synthase (PvCPS15) (red) showing relative abundance. Table S1. GenBank accession numbers for Fig. 2. Table S2. Nucleotide information of gene sequences used in this study. [file PLB-27-698-s001.docx]

Supplementary material for

**Characterization of switchgrass (*Panicum virgatum* L.) PvKSL1 as levopimaradiene/ abietadiene-type diterpene synthase**

Gabrielle Wyatt, Philipp Zerbe, Kira Tiedge

**
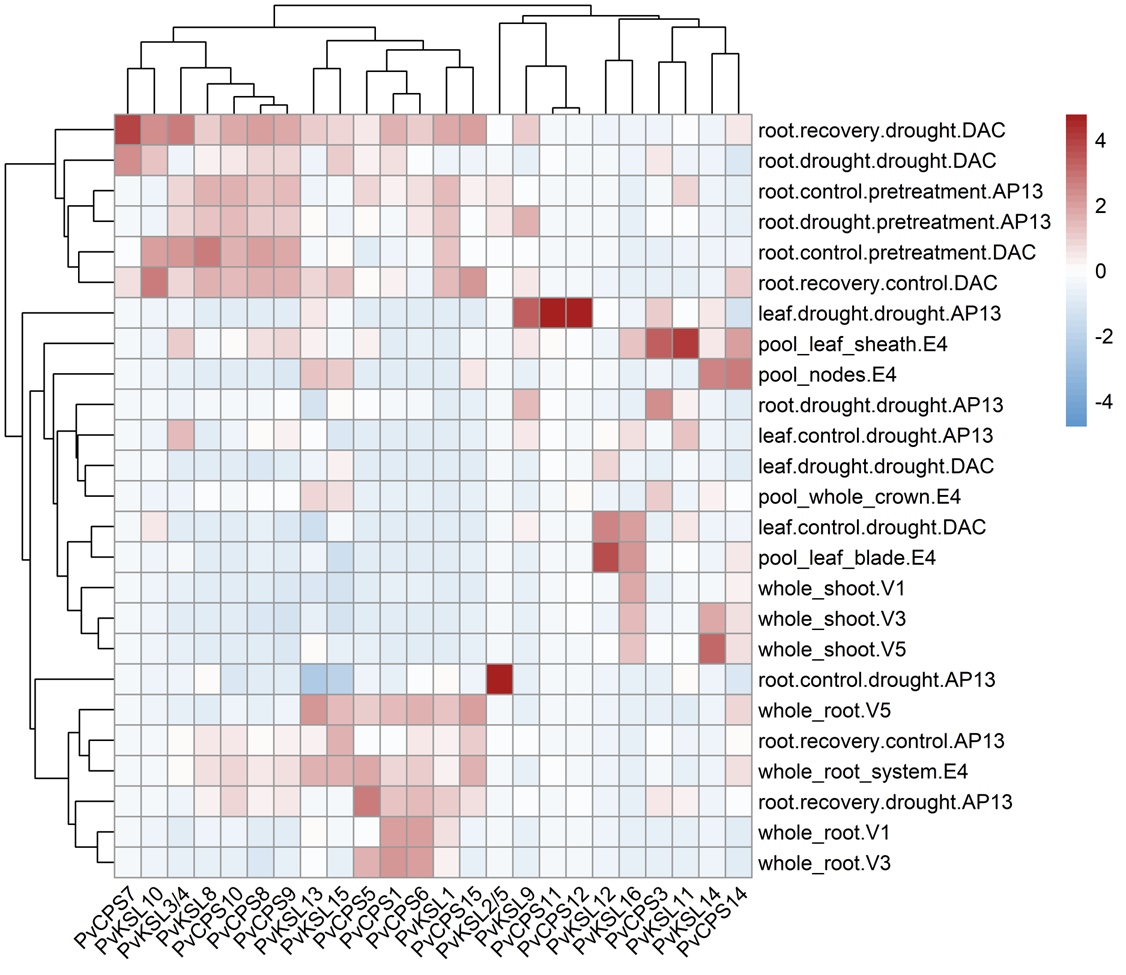
**

**Supplemental Fig S1.** Hierarchical clustering of *Pv*KSL1 with other characterized diTPS using publicly available transcriptome data; all metadata can be found on GeneAtlas ([phytozome-next.jgi.doe.gov/geneatlas](https://phytozome-next.jgi.doe.gov/geneatlas/))

**
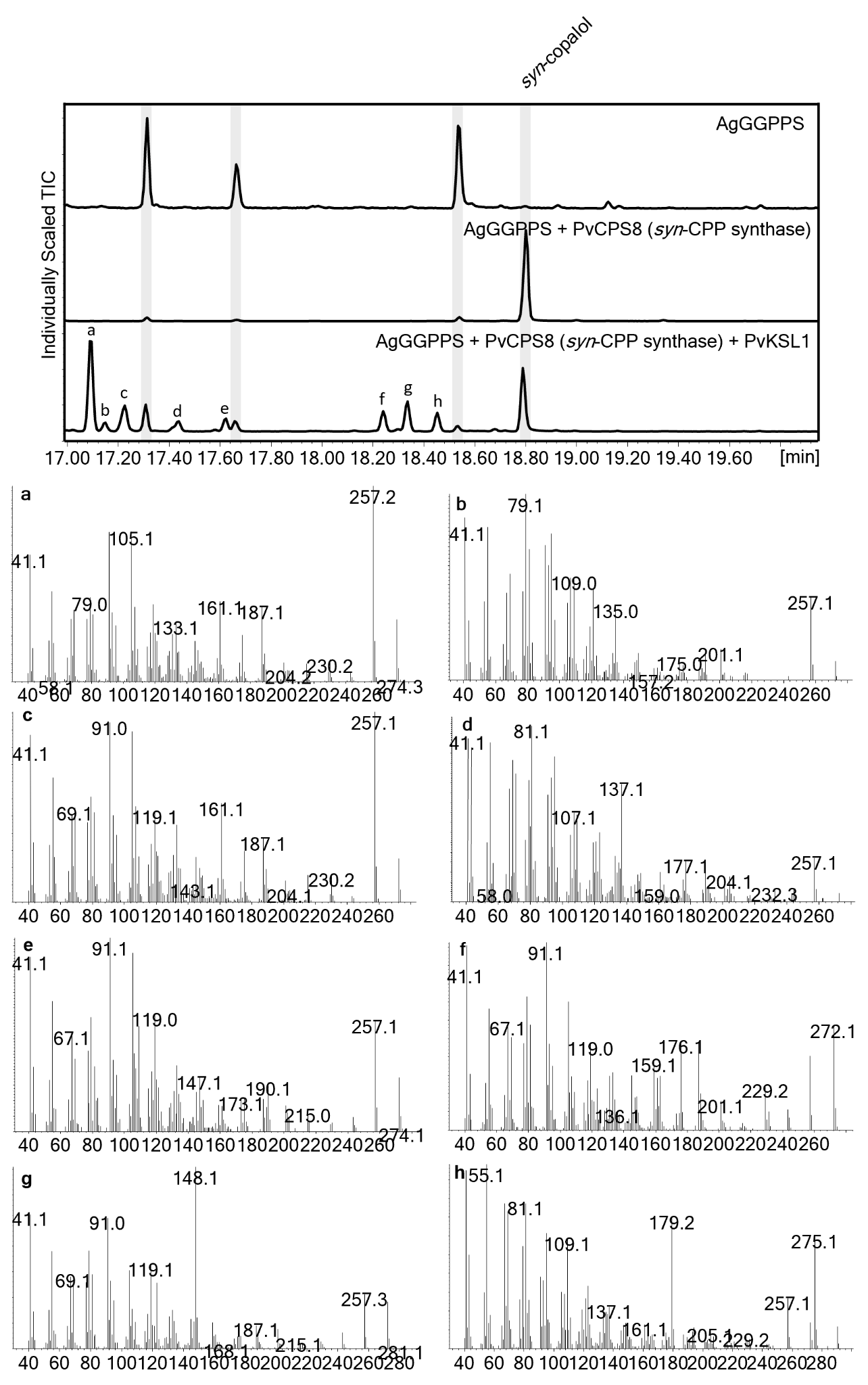
**

**Supplemental Fig S2.** GC-MS chromatogram and mass spectra of product profiles for switchgrass *syn*-CPP synthase (PvCPS8) alone and in combination with PvKSL1, resulting in unknown products (a)-(h).


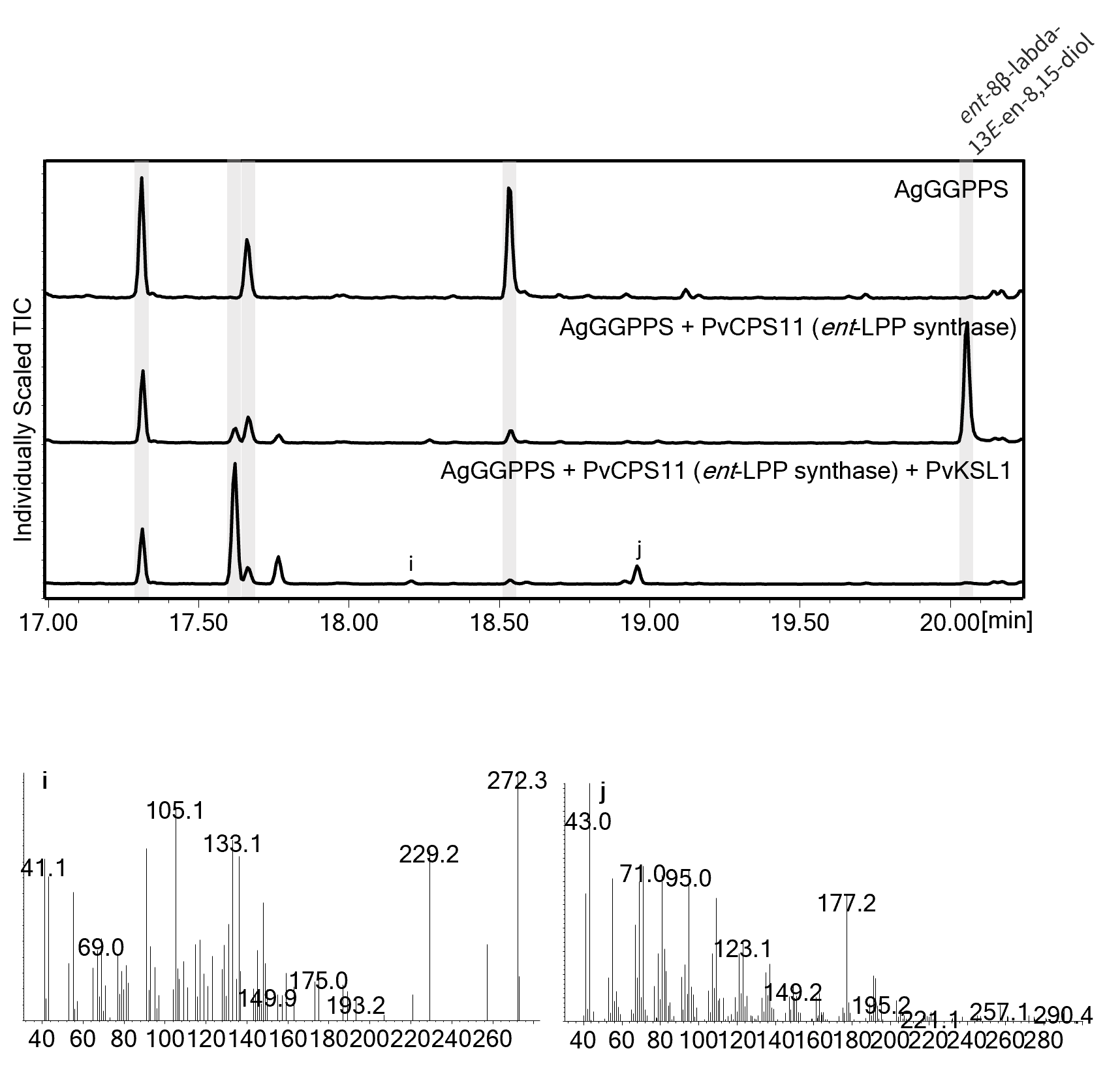


**Supplemental Fig S3.** GC-MS chromatogram and mass spectra of product profiles for switchgrass *ent*-LPP synthase (PvCPS11) alone and in combination with PvKSL1, resulting in unknown products (i) and (j).

**
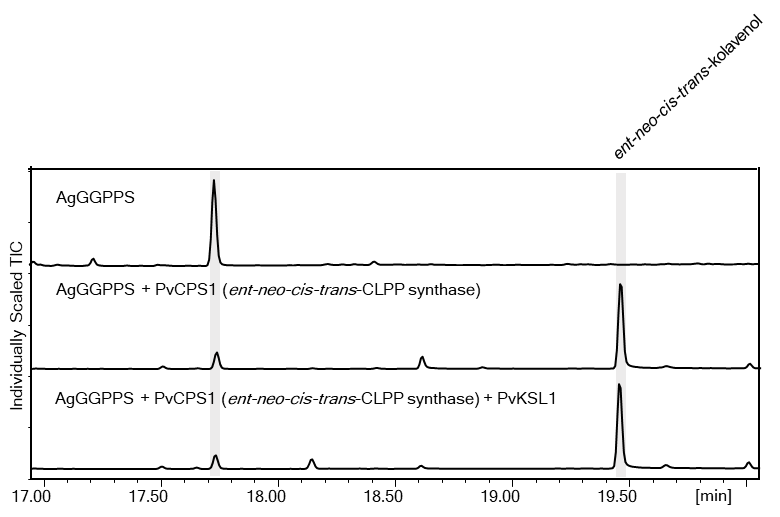
**

**Supplemental Fig S4.** GC-MS chromatogram of product profiles for switchgrass *ent-neo-CT-CLPP* synthase alone and in combination with PvKSL1; no new products resulting


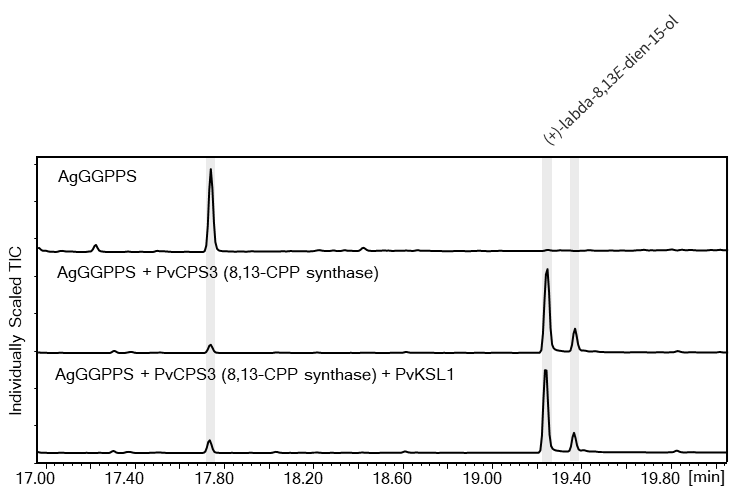


**Supplemental Fig S5.** GC-MS chromatogram of product profiles for switchgrass 8,13-CPP synthase alone and in combination with PvKSL1; no new products resulting.


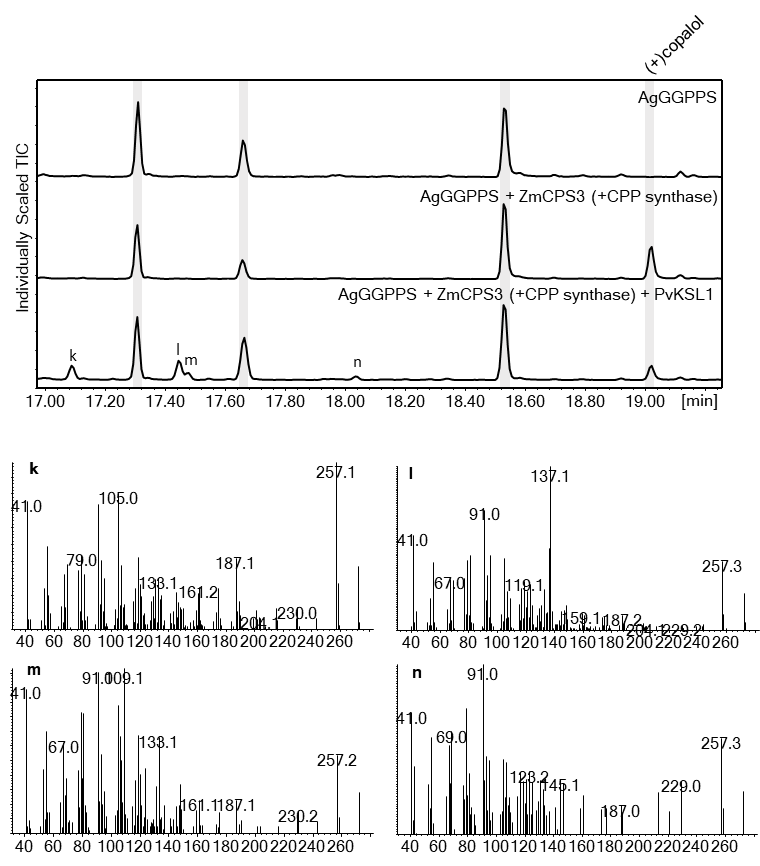


**Supplemental Fig S6.** GC-MS chromatogram and mass spectra of product profiles for *Zea mays* (+)-CPP synthase (*Zm*CPS3) alone and in combination with *Pv*KSL1, resulting in unknown products (k), (l), (m), and (n).

**
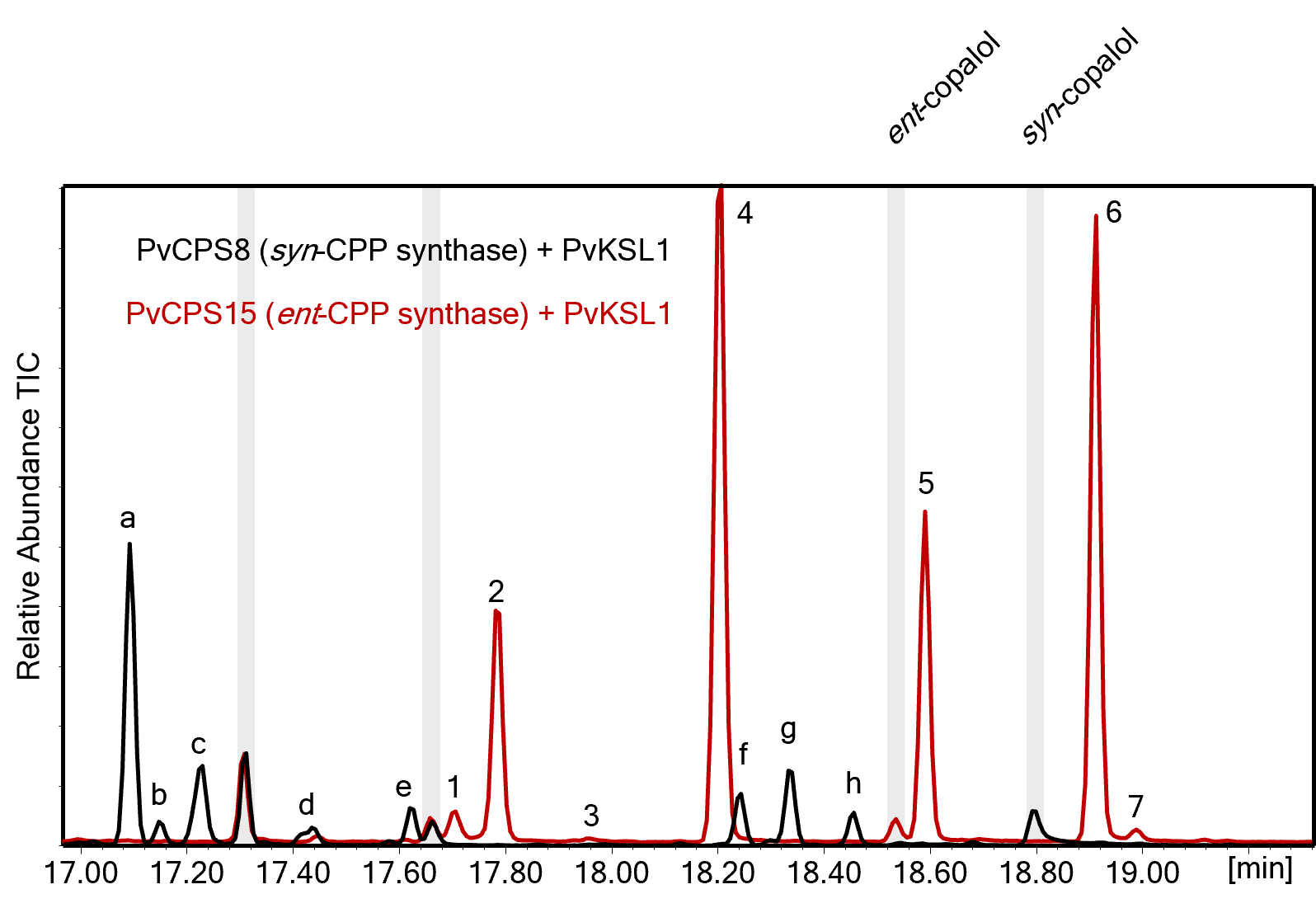
**

**Supplemental Fig S7.** GC-MS chromatogram of product profiles for PvKSL1 with *syn*-CPP synthase (PvCPS8) (black) and *ent*-CPP synthase (PvCPS15) (red) showing relative abundance.

**Supplemental Table S1.** GenBank accession numbers for Fig. 2.

| **Enzyme name** | **GenBank Accession** |  | **Enzyme name (continued)** | **GenBank Accession** |
| --- | --- | --- | --- | --- |
| AgAS | Q38710 |  | PvKSL1 | KAG2634584 |
| EpTPS8 | ALE19952 |  | PvKSL2/5 | AXK78851 |
| EpTPS23 | ALE19953 |  | PvKSL3/4 | AXK78852 |
| GbLPS | Q947C4 |  | PvKSL8 | AXK78855 |
| OmTPS5 | AZB50373 |  | PvKSL13 | AXK78856 |
| OsKS1 | Q0JA82 |  | PvKSL15 | AXK78857 |
| OsKSL4-1 | Q0JEZ8 |  | SiTPS5 | QJA42339 |
| OsKSL5 | Q6Z5J6 |  | SiTPS8 | QJA42341 |
| OsKSL6 | A4KAG8 |  | SiTPS28 | QJA42358 |
| OsKSL7 | Q00G37 |  | SiTPS29 | QJA42359 |
| OsKSL8 | Q6BDZ9 |  | Sobic.005G161600 | KXG28751 |
| OsKSL10 | Q2QQJ5 |  | TaKSL1 | BAL41688 |
| OsKSL11 | Q1AHB2 |  | TaKSL2 | BAL41689 |
| PaISO | Q675L5 |  | TaKSL3 | BAL41690 |
| PaLAS | Q675L4 |  | TaKSL4 | BAL41691 |
| PbLAS1 | M4HXU6 |  | TaKSL5 | BAL41692 |
| PbMISO1 | M4HXW5 |  | TaKSL6 | BAL41693 |
| PbMPIM1 | M4HY08 |  | ZmKSL2 | ONM14042 |
| PcMISO1 | M4HYP3 |  | ZmKSL3 | ONM14041 |
| PcMPIM1 | M4HY08 |  | ZmKSL4 | ONM32348 |
| PhHAL.3G396600 | PAN20858 |  | ZmKSL5 | ONM14042 |
| PpCPSKS | A5A8G0 |  |  |  |

**Supplemental Table S2.** Nucleotide information of gene sequences used in this study

| **Gene/ Mutation** | **Nucleotide sequence** |
| --- | --- |
| Pavir.2NG177700  n-terminally truncated | ATGTTACCACACCGGCAAAAAAGGAATTTGGAGAATGGAATAACGGAGAAGCTCCTACTCACCAAACAGTGTCCATCAACCTATGACACGGCTTGGGTTAGTATGGTACCAGCGCAAGGCTCTCCTCAGACACCACGGTTTCCACAATTTGTGCAGTGGATCCAACAAAACCAAAACGATGATGGTTCTTGGGGCCTTGGCAACCTCGACCTGAGTTTGCTTGGCAAGGATGCCATCACCTCAACAACGGCTTGCATTCTTGCCCTCAAGAGATGGAGCATCGGGAATGAGCAGATCATGAAAGGGCTCCATTTTATCAGAGAAAATTATTCCAGGATAAAGGATGAGAACTGTTTTTCACCCGTAGGGTTCAACCTCATCTTCCCAAGAATGATAACGATCGGTATAGATATGGGCTTGGAATTTCCGCTTAGTGAATCTGATATTGATTGGGTATCAGGACTTCGAGAAATGGAATTATTAAGGCACGACAGTGTTGAGGCTTCTAGACGAAATGGTTATTATATGGCATATGTAGCAGAAGGATTAGGAGACATGCAAGAAATAAATCAGGCCCTGATGTATCAAAGAAAGAATGGATCCTTATTTAACTCGCCTGCTGCAACTGCTGCTGCAGCAATCCACACCCAAGATCCTGGTTCTCTCATGTACTTGGATTTCCTTGCAGAAAAGTGTAGCAGCTCAGTTCCAACTGTGTATCCAATGGACATATATTCCCAACTTTGCTTGGTAGACACCCTTGAAAAGATTGGAATTGCCCACCATTTTCCTTCAGAGATAAAACACATAGTGAATATGGTATACAGATCCTGGTTGGAAAAGGATGAAGAGATCATAATGGACATGGAGACTTGCGCAATGGCATTCCGCATCCTGCGCGTGCATGGATATGACATCTCATCTGATGCACTTTCTCACCTTGCTCACCAGGTTTTAAGGTTCAACAGTTCAGTAAGTGATGATGTAAACAATGCAAAGGCTTTGCTTGAATTATACAAAGCTTCACGGATACGCATATTTGAAGACGAGTGGTCTCTGGACAATATTGAATCATGTACAAGAAAACTGCTGAACCAGCAATTTTGCTCCAGGAAATTTCAAGGATCACGAACGCTCCAAGAGGGAGAGTATGGTCTTAAATTCCCATTCTACCCTGGCACATTGGAACCAGCTCAGCAGAAGTGGACCATAGAGCGATACGACATCAAGCATGTCCAGATGCGAAAATCAGCATTCATGGCACCTCACTCAGATGAACGTTTTCTAACATTAGCCATTGAAGAGTTCCGTTCCTCCCAATCGGTATACCAGGAAGAACTTGCTTGCATTAAGAGATGGGCGGAAGAGATTGGATTGCACCAGCTCAAGTTCGCCAGAGTTATGCCACTGGATGTCTTTGTCTTCATGGCTTCCACTGTGTTTGCTCCTGAACTATATGACGCCAGCATTGCGTGGATGAAGAATAGTATTCTTACGACGGTAGTTGATGACTTCTTCGAAAACCAAGGATCTATAGAAGAGTTAAAGAACCTTGTTGCACTAATTGAAAAATGGGACGCACATGAAGAGGTTGGGTTCTGCTCTGAGAACGTAGAAGCTTTATTTTATGCAGTTTACAGCACCAACAGCCAAATAGGGGCAAAGGCAGAAGAGATACAGAACCGGAGCATCATGTGCCACATTGCTGAAGTTTGGTCCGATGTGGTGAGGGCGTACATGATCGAGCAAGAGTGGACACGGGAAAGGCACGTACCGACCATGCAAGAGTACATGTGTGCCGCCGAAGTAAGCATTGCATTGGGTGCTATCGTTGCCCCATCGTTGTACTTGGTCGGACCAAAGCTTTCGGAAGGCATGATAAGGAGTTCGGAGTACAAGGATCTGCTTAGACATATGCGTACCAGCGTTCGCTTCCTGAACGATCTGGGCACTTACAAAAAGGAGATGACTCACGGATGCATCAACAGCGTCCTGCTGAAAGCACGTGTTAGCGACCTGTCGATGTCACCAGCGTCCATCGAAACTGCCAAGACGGGCATTTGTGAGGCCATTGCTGACTCTCAGAGGGAGCTGTTGCGGCTGGTGCTTAGGGATGGGGGTCCAATTCCTAGGCTGTGCAGGGACATTTTCTGGAATACCTACAGAATAGGACAACGGTTCTACTCGCAGGGAGACGGCTTTGGCATGCCGCAAGAATTGGTGGCGGCGGTGAATGCGGTGGTTCATGAGCCACTTAATAAAGAGGTAATGCTTCCACCCCGCAAGAAAAGAGGGTATTCTTCAGCACTGGTGTGA |
| A660S sense | AGTAAGCATTagtTTGGGTGCTATC |
| A660S anti-sense | TCGGCGGCACACATGTAC |
| W626L sense | TGCTGAAGTTctgTCCGATGTGG |
| W626L anti-sense | ATGTGGCACATGATGCTC |
| I664V sense | ATTGGGTGCTgtaGTTGCCCCAT |
| I664V anti-sense | GCAATGCTTACTTCGGCG |
| I664L sense | ATTGGGTGCTctgGTTGCCCCATC |
| I664L anti-sense | GCAATGCTTACTTCGGCG |
| Y663H sense | GGTGAGGGCGcacATGATCGAGC |
| Y663H anti-sense | ACATCGGACCAAACTTCAGC |
| Quad I664L sense | TTGGGTGCTctgGTTGCCCCATC |
| Quad I664L anti-sense | ACTAATGCTTACTTCGGCG |
